# Stage-Specific Survival in Breast Cancer in Chinese and White Women: Comparative Data Analysis

**DOI:** 10.2196/40386

**Published:** 2022-11-15

**Authors:** Jun Wang, Juan Zhou, Lei Liu, San-Gang Wu

**Affiliations:** 1 Department of Head and Neck Oncology, Cancer Center West China Hospital Sichuan University Chengdu China; 2 Department of Radiation Oncology, State Key Laboratory of Biotherapy West China Hospital Sichuan University Chengdu China; 3 Department of Obstetrics and Gynecology, the First Affiliated Hospital of Xiamen University School of Medicine Xiamen University Xiamen China; 4 Department of Radiation Oncology the First Affiliated Hospital of Xiamen University School of Medicine, Xiamen University Xiamen China; 5 Xiamen Key Laboratory of Radiation Oncology Xiamen Cancer Center Xiamen China

**Keywords:** breast cancer, AJCC, American Joint Committee on Cancer, Chinese, White American, survival, surveillance, epidemiology, staging, pathological prognostic staging, AJCC stage, overall survival, cancer-specific survival

## Abstract

**Background:**

Stage-specific survival, according to the eighth edition of the American Joint Committee on Cancer (AJCC) pathological prognostic staging (PPS) on breast cancer (BC), between Chinese and White American women remains unclear.

**Objective:**

This study aimed to assess stage-specific survival in BC between Chinese and White American women according to the eighth AJCC PPS.

**Methods:**

We included Chinese and White American women with BC diagnosed between 2010 and 2018 from the Surveillance, Epidemiology, and End Results database. A chi-square test, the Kaplan–Meier method, a receiver operating characteristic (ROC) curve, and multivariate Cox proportional hazards models were used for data analysis.

**Results:**

We included 376,818 individuals in this study: 369,522 White American and 7296 Chinese. Of them, 149,452 (39.7%) migrated from the seventh AJCC anatomic staging (AS) to the eighth AJCC PPS, 22,516 (6.0%) were upstaged, and 126,936 (33.7%) were downstaged. With a median follow-up duration of 44 months, the 5-year overall survival and cancer-specific survival (CSS) for the entire group were 87.4% and 95.9%, respectively. The seventh AJCC AS (*P*<.001) and the eighth AJCC PPS (*P*<.001) could significantly predict the survival outcomes of BC, and multivariate analysis revealed that both staging systems were significant prognostic indicators of CSS. The ROC curve revealed that the PPS had a better discriminating ability than the AS (area under the curve [AUC] 0.769 vs 0.753, *P*<.001). Similar trends were observed after stratification by the 2 ethnic groups. The eighth AJCC PPS had better discriminating ability than the seventh AJCC AS among both White American (AUC 0.769 vs 0.753, *P*<.001) and Chinese patients (AUC 0.790 vs 0.776, *P*<.001). In the seventh AJCC AS, Chinese women had better CSS in stage IA (*P*=.02), stage IIA (*P*=.005), and stage IIIB (*P*=.04) disease than White American women, but no significant CSS was observed in stage IB, IIB, IIIA, and IIIC disease between the 2 ethnic groups. Regarding the eighth AJCC PPS, Chinese women had better CSS in stage IA (*P*=.002) and IIIA (*P*=.046) disease than White American women, and CSS was similar in Chinese and White American women in other substages.

**Conclusions:**

The eighth AJCC PPS has a similar discriminative ability between White American and Chinese individuals with BC compared with the seventh AJCC AS. Therefore, the eighth AJCC PPS is also applicable to Chinese individuals with BC.

## Introduction

Breast cancer (BC) is the most commonly diagnosed cancer among women, and 2,261,419 new cases were estimated to occur worldwide in 2020 [1]. The incidence of BC varies among different countries and racial and ethnic groups with age-adjusted rates of 129 per 100,000 population for White American and 75.1 per 100,000 population for Chinese women in the United States, while the rate was 30.69 per 100,000 population for Chinese women based on the Chinese population [2,3]. The survival outcomes are also discrepant in different races: White American individuals with BC have better cancer-specific survival (CSS) than African American individuals but poorer survival than Chinese individuals [4,5].

Traditional American Joint Committee on Cancer (AJCC) anatomic staging (AS) of BC includes tumor size (T), lymph nodes (N), and distant metastasis (M), which has been extensively used for predicting prognosis and guiding treatment in BC [6,7]. The eighth edition of the AJCC pathological prognostic staging (PPS) integrates the estrogen receptor (ER), progesterone receptor (PR), human epidermal growth factor-2 (HER2), and tumor grade into AS, which facilitates better precise prognostic stratification than AS [8-10]. However, the vast majority of the included patients for establishing the initial model of the eighth AJCC PPS were White American and Hispanic American [11], and whether the new PPS is also applicable to the Chinese individuals with BC remains unclear. As the country with the largest population worldwide, China has approximately 400,000 new BC cases annually, and previous studies have shown that Chinese patients have different morbidity and survival outcomes than White American patients [3,4,12]. Therefore, more ethnic-based studies are needed to explore the value of the PPS in Chinese individuals with BC to make this new staging more widely available.

Several studies have attempted to validate the value of the new staging in the Chinese population [13-15]. However, these studies included specific subgroups, such as T1-2N1 and triple-negative breast cancer (TNBC), which could not represent patients with BC in general [13-15]. In addition, the small sample size and the heterogeneity of treatment also made it difficult to accurately assess the value of the new staging in Chinese patients. In this study, we used a population-based cohort to compare stage-specific survival in BC between White American and Chinese women in accordance with the eighth AJCC PPS, and to expand the applicability of the new staging system in more races.

## Methods

### Data Source and Patient Selection

The patient data in this study were extracted from the Surveillance, Epidemiology, and End Results (SEER) database between 2010 and 2018. The SEER database collects information on cancer statistics, treatment, and survival, covering approximately 48% of the population in the United States [16]. Patients with the following criteria were included: (1) pathologically diagnosed with invasive BC; (2) White American or Chinese individuals with BC; (3) having detailed information on age, TNM stage, ER status, PR status, HER2 status, histology subtype, tumor grade, surgery, radiotherapy, and chemotherapy administration. Male patients, patients with contralateral BC, and those diagnosed with a distant metastatic stage were excluded from this study.

### Variables and Endpoints

We selected the variables including age (<50, 50-70, and >70 years), race and ethnicity (White American and Chinese), grade (well-, moderately-, poorly differentiated, and undifferentiated), histological subtype (infiltrating duct carcinoma, lobular carcinoma, mixed, and other carcinomas), molecular subtype (luminal A, luminal B, HER2-enriched, and triple-negative BC), T stage (T0-T4), N stage (N0-N3), and the seventh and eighth AJCC staging (IA-IIIC). The races and ethnicities of White American (code 01) and Chinese (code 04) were chosen for analysis using “Race/ethnicity” codes in the SEER database. The end point of this study was CSS, which was calculated as the time from BC diagnosis to the occurrence of BC-related death.

### Ethical Considerations

This study was approved by the ethics committee of the First Affiliated Hospital of Xiamen University (Xiamen) and West China Hospital, Sichuan University (Chengdu; 2021GGB027). Informed consent is not required because the data were extracted from the SEER database after obtaining permission from the administrator. In addition, the privacy of the participants was well protected through anonymization and deidentification of their information.

### Statistical Analysis

The Pearson chi-square test was used to compare the differences in baseline characteristics and stage migration changes between groups of Chinese and White American individuals with BC. The receiver operating characteristic (ROC) curve was used to identify the discriminating ability of the AS and PPS. The Kaplan–Meier method was used to plot the survival curves, and the log-rank test was used to compare the differences. Multivariate Cox proportional hazards models were used to calculate the independent risk predictors of CSS. Sensitivity analyses were used to investigate the effect of race on CSS after stratification by different AJCC substages. SPSS (version 22.0; IBM Corp) was used for analyzing all the data. A *P* value less than .05 was defined as the threshold for statistical significance.

## Results

### Cohort Characteristics

In total, 376,818 individuals with BC were identified in this study. Of them, 369,522 (98.1%) were White American and 7296 (1.9%) were Chinese. The proportions of patients aged <50, 50-70, and ≥70 years were 18.3% (n=68,833), 50.9% (n=191,831), and 30.8% (n=116,154), respectively. The majority of the patients had infiltrating ductal carcinoma (n=294,359, 78.1%), a low-grade tumor (n=279,429, 74.2%), luminal A subtype BC (n=288,545, 76.6%), T1-2 stage BC (n=347,931, 92.3%), and N0-1 stage BC (n=351,666, 93.3%).

Regarding the distribution of baseline characteristics between White American and Chinese individuals, the latter were more likely to be younger (*P*<.001) and have a lower-grade tumor (*P*<.001), infiltrating duct carcinoma (*P*<.001), luminal B or HER2-enriched subtype (*P*<.001), and a lower-stage tumor (*P*<.001) than White American participants. Among all participants, stage IA, IB, IIA, IIB, IIIA, IIIB, and IIIC disease accounted for 53.3% (n=200,926), 2.3% (n=8,569), 23.0% (n=86,651), 11.1% (n=41,837), 5.9% (n=22,237), 2.0% (n=7649), and 2.4% (n=8949) of cases in the seventh AJCC AS and for 66.1% (n=249,173), 15.5% (n=58,405), 7.8% (n=29,379), 3.2% (n=12,116), 3.9% (n=14,800), 1.9% (n=7175), and 1.5% (n=5770) of cases in the eighth AJCC PPS, respectively.

With regard to the treatments, 96.1% (n=362,189) of the participants received surgical intervention, 52.7% (n=198,399) of them received radiotherapy, and 36.7% (n=139,160) of them received chemotherapy. White American participants were more likely to undergo breast-conserving surgery, while more Chinese participants were treated with mastectomy (*P*<.001). In addition, Chinese participants were more prone to receiving chemotherapy (*P*<.001), while White American patients were more likely to be treated with radiotherapy (*P*<.001). Detailed information on the study population is presented in Table 1.

**Table 1:** Participants’ characteristics (N=376,818).

Variables			Total, n (%)	Race and ethnicity, n (%)			*P* value				
				White American (n=369,522)	Chinese (n=7296)						
**Age groups (years)**								<.001			
	<50		68,833 (18.3)	66,803 (18.1)	2030 (27.8)						
	50-69		191,831 (50.9)	188,051 (50.9)	3780 (51.8)						
	≥70		116,154 (30.8)	114,668 (31.0)	1486 (20.4)						
**Grade**								<.001			
		Well differentiated	97,389 (25.8)	95,820 (25.9)	1569 (21.5)						
		Moderately differentiated	173,649 (46.1)	170,182 (46.1)	3467 (47.5)						
		Poorly differentiated or undifferentiated	105,780 (28.1)	103,520 (28.0)	2260 (31.0)						
**Histology**								<.001			
		Infiltrating duct carcinoma	294,359 (78.1)	288,243 (78.0)	6116 (83.8)						
		Lobular carcinoma	39,292 (10.4)	38,880 (10.5)	412 (5.6)						
		Mixed	22,473 (6.0)	22,186 (6.0)	287 (3.9)						
		Other	20,694 (5.5)	20,213 (5.5)	481 (6.6)						
**Molecular subtype**								<.001			
		Luminal A	288,545 (76.6)	283,251 (76.7)	5294 (72.6)						
		Luminal B	37,325 (9.9)	36,449 (9.9)	876 (12.0)						
		HER2-enriched	14,065 (3.7)	13,611 (3.7)	454 (6.2)						
		Triple-negative	36,757 (9.8)	36,086 (9.8)	671 (9.2)						
**T (tumor size) stage**								<.001			
		T0	164 (0.0)	160 (0.0)	4 (0.0)						
		T1	237,926 (63.1)	233,569 (63.2)	4357 (59.7)						
		T2	110,005 (29.2)	107,560 (29.1)	2445 (33.5)						
		T3	19,784 (5.3)	19,443 (5.3)	341 (4.7)						
		T4	8939 (2.4)	8790 (2.4)	149 (2.0)						
**N (lymph nodes) stage**								.63			
		N0	272,990 (72.4)	267,686 (72.4)	5304 (72.7)						
		N1	78,676 (20.9)	77,149 (20.9)	1527 (20.9)						
		N2	16,203 (4.3)	15,895 (4.3)	308 (4.2)						
		N3	8949 (2.4)	8792 (2.4)	157 (2.2)						
**Seventh version of the American Joint Committee on Cancer (AJCC) staging**								<.001			
		IA	200,926 (53.3)	197,231 (53.4)	3695 (50.6)						
		IB	8569 (2.3)	8422 (2.3)	147 (2.0)						
		IIA	86,651 (23.0)	84,765 (22.9)	1886 (25.8)						
		IIB	41,837 (11.1)	40,962 (11.1)	875 (12.0)						
		IIIA	22,237 (5.9)	21,827 (5.9)	410 (5.6)						
		IIIB	7649 (2.0)	7523 (2.0)	126 (1.7)						
		IIIC	8949 (2.4)	8792 (2.4)	157 (2.2)						
**Eighth version of the AJCC staging**								<.001			
		IA	249,173 (66.1)	244,477 (66.2)	4696 (64.4)						
		IB	58,405 (15.5)	57,236 (15.5)	1169 (16.0)						
		IIA	29,379 (7.8)	28,722 (7.8)	657 (9.0)						
		IIB	12,116 (3.2)	11,843 (3.2)	273 (3.7)						
		IIIA	14,800 (3.9)	14,538 (3.9)	262 (3.6)						
		IIIB	7175 (1.9)	7023 (1.9)	152 (2.1)						
		IIIC	5770 (1.5)	5683 (1.5)	87 (1.2)						
**Surgery**								<.001			
		No surgery	14,145 (3.8)	13,867 (3.8)	278 (3.8)						
		Breast-conserving surgery	213,329 (56.6)	209,671 (56.7)	3658 (50.1)						
		Mastectomy	148,860 (39.5)	145,509 (39.4)	3351 (45.9)						
		Unknown	484 (0.1)	475 (0.1)	9 (0.1)						
**Radiotherapy**								<.001			
		No	167,183 (44.4)	163,677 (44.3)	3506 (48.1)						
		Beam radiation	187,640 (49.8)	184,138 (49.8)	3502 (48.0)						
		Radioactive implants	10,759 (2.9)	10,631 (2.9)	128 (1.80)						
		Unknown	11,236 (3.0)	11,076 (3.0)	160 (2.2)						
**Chemotherapy**								<.001			
		No	237,658 (63.1)	233,283 (63.1)	4375 (60.0)						
		Yes	139,160 (36.9)	136,239 (36.9)	2921 (40.0)						

### Stage Migration

According to the seventh AJCC AS, 197,231 (53.4%), 8422 (2.3%), 84,765 (22.9%), 40,962 (11.1%), 21,827 (5.9%), 7523 (2.0%), and 8792 (2.4%) White American participants versus 3695 (50.6%), 147 (2.0%), 1886 (25.8%), 875 (12.0%), 410 (5.6%), 126 (1.7%), and 157 (2.2%) Chinese participants had stage IA, IB, IIA, IIB, IIIA, IIIB, and IIIC disease, respectively (*P*<.001). According to the eighth AJCC PPS, 244,477 (66.2%), 57,236 (15.5%), 28,722 (7.8%), 11,843 (3.2%), 14,538 (3.9%), 7023 (1.9%), and 5683 (1.5%) White American participants versus 4696 (64.4%), 1169 (16.0%), 657 (9.0%), 273 (3.7%), 262 (3.6%), 152 (2.1%), and 87 (1.2%) Chinese participants had stage IA, IB, IIA, IIB, IIIA, IIIB, and IIIC disease, respectively (*P*<.001; Table 1). A total of 149,452 (39.7%) participants migrated from the seventh AJCC AS to the eighth AJCC PPS (n=22,516, 6.0% upstaged and n=126,936, 33.7% downstaged). Among the upstaged participants, 22,127 (6.0%) were White American and 389 (6.3%) were Chinese, while among the downstaged participants, 124,368 (33.6%) were White American and 2586 (35.2%) were Chinese (*P*=.004). Furthermore, the disease stages of 223,027 (60.4%) White American and 4339 (59.5%) Chinese participants remained unchanged. There was a significant difference in stage migration (upstaging, downstaging, and unchanging stage) between White American and Chinese participants (*P*=.004). The frequencies of stage discrepancies between White American and Chinese participants are shown in Table 2.

**Table 2:** The frequencies of stage discrepancies between White American and Chinese participants.

Seventh AJCC^a^ AS^b^			Eighth AJCC PPS^c^, n (%)															Total, n (%)
			IA		IB		IIA		IIB		IIIA		IIIB		IIIC			
**White American**																		
	IA	183,587 (49.7)		13,644 (3.7)		0 (0)		0 (0)		0 (0)		0 (0)		0 (0)		197,231 (53.4)		
	IB	7984 (2.2)		438 (0.1)		0 (0)		0 (0)		0 (0)		0 (0)		0 (0)		8422 (2.3)		
	IIA	48,507 (13.1)		14,764 (4.0)		21,494 (5.8)		0 (0)		0 (0)		0 (0)		0 (0)		84,765 (22.9)		
	IIB	4399 (1.2)		17,924 (4.9)		6058 (1.6)		8379 (2.3)		4202 (1.1)		0 (0)		0 (0)		40,962 (11.1)		
	IIIA	0 (0)		10,466 (2.8)		1170 (0.3)		3464 (0.9)		4245 (1.1)		399 (0.1)		2083 (0.6)		21,827 (5.9)		
	IIIB	0 (0)		0 (0)		0 (0)		0 (0)		2641 (0.7)		3083 (0.8)		1799 (0.5)		7523 (2.0)		
	IIIC	0 (0)		0 (0)		0 (0)		0 (0)		3450 (0.9)		3541 (1.0)		1801 (0.5)		8792 (2.4)		
	Total	244,477 (66.2)		57,236 (15.5)		28,722 (7.8)		11,843 (3.2)		14,538 (3.9)		7023 (1.9)		5683 (1.5)		369,522 (100)		
**Chinese**																		
	IA	3447 (47.2)		248 (3.4)		0 (0)		0 (0)		0 (0)		0 (0)		0 (0)		3695 (50.6)		
	IB	143 (2.0)		4 (0.1)		0 (0)		0 (0)		0 (0)		0 (0)		0 (0)		147 (2.0)		
	IIA	1040 (14.3)		350 (4.8)		496 (6.8)		0 (0)		0 (0)		0 (0)		0 (0)		1886 (25.8)		
	IIB	66 (0.1)		404 (5.5)		133 (1.8)		194 (2.7)		78 (1.1)		0 (0)		0 (0)		875 (12.0)		
	IIIA	0 (0)		163 (2.2)		28 (0.4)		79 (1.1)		99 (1.4)		9 (0.1)		32 (0.4)		410 (5.6)		
	IIIB	0 (0)		0 (0)		0 (0)		0 (0)		38 (0.5)		66 (0.9)		22 (0.3)		126 (1.7)		
	IIIC	0 (0)		0 (0)		0 (0)		0 (0)		47 (0.6)		77 (1.1)		33 (0.5)		157 (2.2)		
	Total	4696 (64.4)		1169 (16.0)		657 (9.0)		273 (3.7)		262 (3.6)		152 (2.1)		87 (1.2)		7296 (100)		

^a^AJCC: American Joint Committee on Cancer.

^b^AS: anatomic staging.

^c^PPS: pathological prognostic staging.

### Survival and Prognostic Analyses by Race and Ethnicity

**Figure 1:**
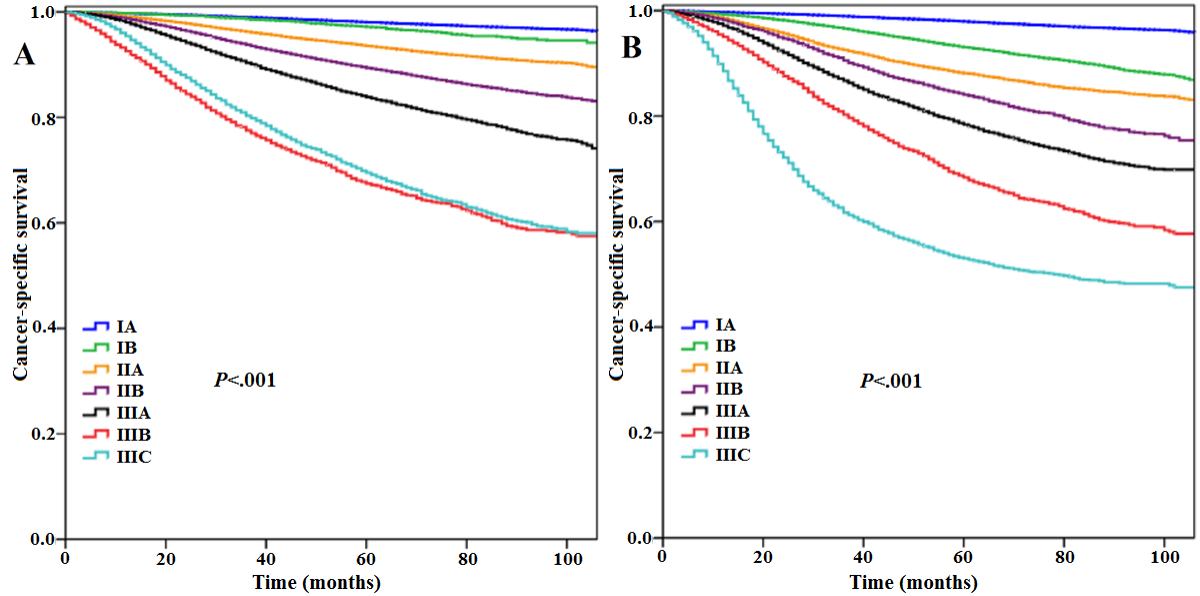
Cancer-specific survival according to the anatomic staging (A) and pathological prognostic staging (B) systems for the entire cohort.

**Figure 2:**
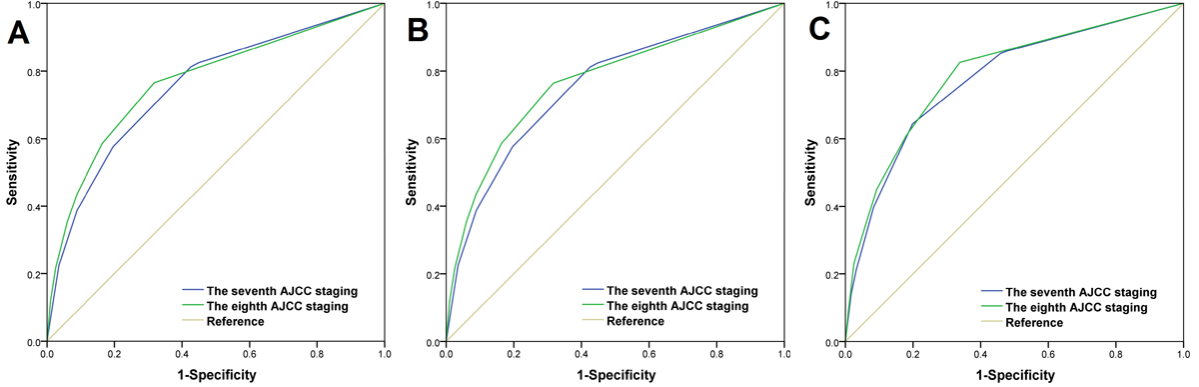
Receiver operating characteristic curve for predicting the discriminating value of the seventh and the eighth editions of the American Joint Committee on Cancer (AJCC) staging system in the entire cohort (A), White American women (B), and Chinese women (C).

With a median follow-up duration of 44 months (range 0-107 months), 42,522 deaths and 17,807 breast cancer–related deaths occurred. The Kaplan–Meier curves showed that 5-year overall survival and CSS for the entire group were 87.4% and 95.9%, respectively. The seventh AJCC AS (*P*<.001; Figure 1A) and the eighth AJCC PPS (*P*<.001; Figure 1B) could significantly predict the survival outcome of BC. Multivariate Cox proportional hazards analysis revealed that the AS and PPS both had significant prognostic predicting value in the study population (Table 3). Kaplan–Meier curves (Figure 1) and ROC curve (area under the curve [AUC] 0.769 vs 0.753, *P*<.001; Figure 2A) indicated that the eighth AJCC PPS had better discriminating ability than the seventh AJCC AS.

**Figure 3:**
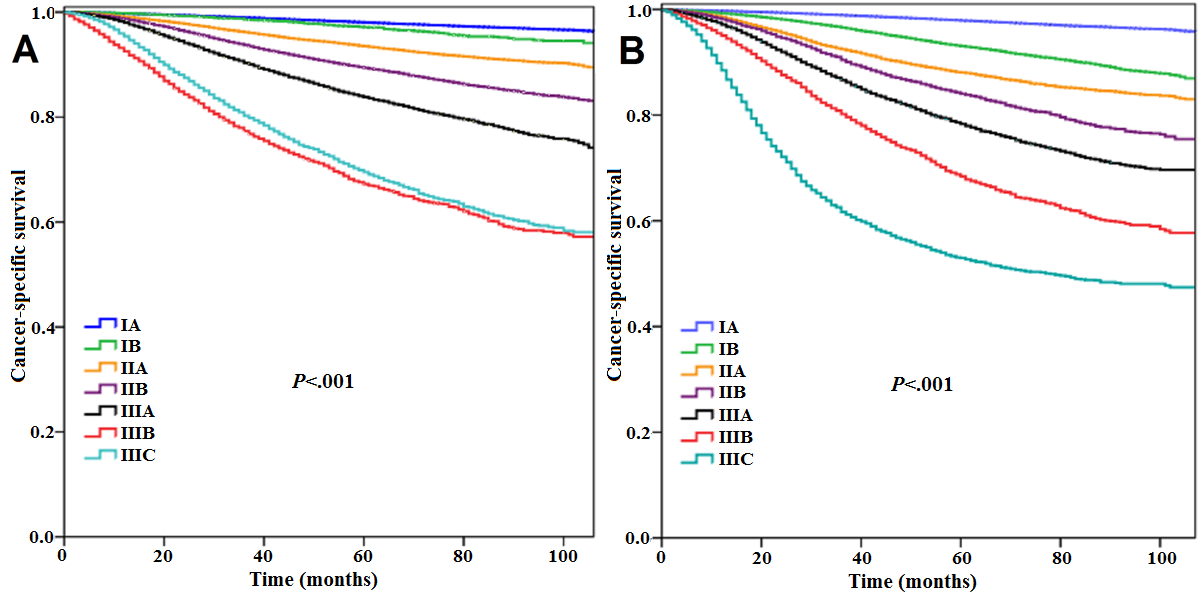
Kaplan–Meier curve for cancer-specific survival according to the seventh (A) and eighth editions of the American Joint Committee on Cancer (B) staging system in White American women.

**Figure 4:**
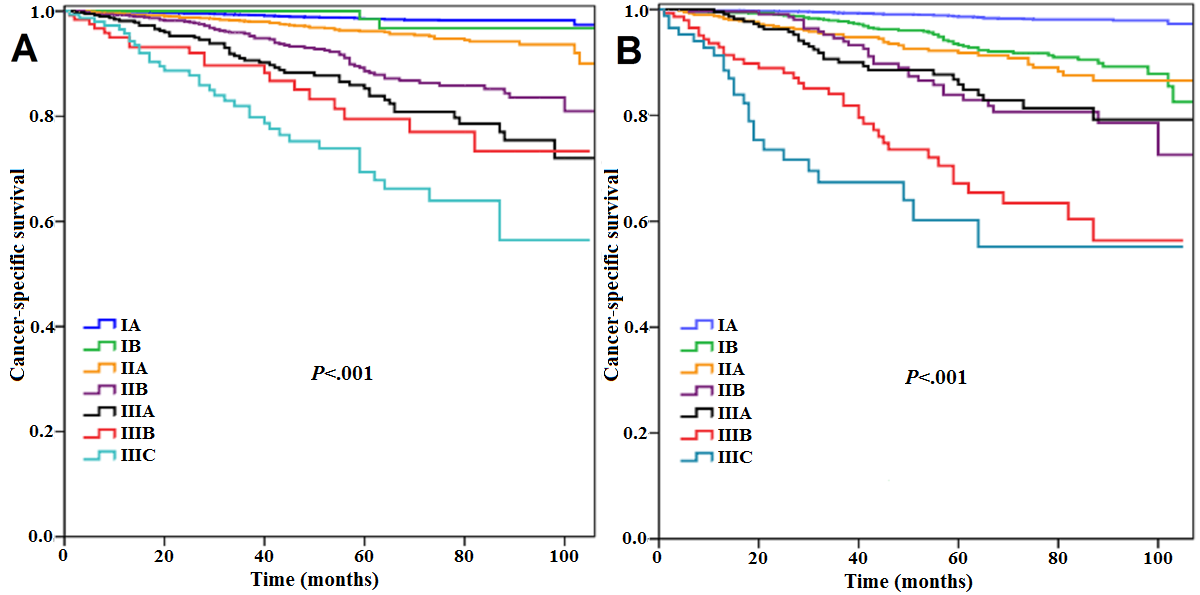
Kaplan–Meier curve for cancer-specific survival according to the seventh (A) and eighth editions of the American Joint Committee on Cancer (B) staging system in Chinese women.

In White American participants, the AJCC AS (*P*<.001; Figure 3A) and the AJCC PPS (*P*<.001; Figure 3B) could also significantly predict survival and prognosis consistent with stages, and multivariate analysis showed that the 2 staging systems were significant prognostic predictors of CSS (Table 4). In addition, the eighth AJCC PPS had better discriminating ability than the seventh AJCC AS in White American participants (AUC 0.769 vs 0.753, *P*<.001; Figure 2B). Similar results were obtained for Chinese participants, in that the seventh AJCC AS (*P*<.001; Figure 4A) and the eighth AJCC PPS (*P*<.001; Figure 4B) both had significant prognostic values (Table 4), and the eighth AJCC PPS still showed better discriminating ability (AUC 0.790 vs 0.776, *P*<.001; Figure 2C) in this population.

**Table 3:** Cox multivariate analysis for cancer-specific survival according to different staging systems.

Variables			Cancer-specific survival						
			Hazard ratio (95% CI)		*P* value		Hazard ratio (95% CI)		*P* value
**Age (year)**									
	<50	1 (reference)		N/A^a^		1 (reference)		N/A	
	50-69	1.115 (1.070-1.162)		<.001		1.100 (1.055-1.146)		<.001	
	≥70	2.458 (2.357-2.564)		<.001		2.413 (2.314-2.517)		<.001	
**Race and ethnicity**									
	White	1 (reference)		N/A		1 (reference)		N/A	
	Chinese	0.827 (0.732-0.934)		.002		0.827 (0.732-0.934)		.002	
**Histology**									
	Infiltrating duct carcinoma	1 (reference)		N/A		1 (reference)		N/A	
	Lobular carcinoma	1.037 (0.984-1.093)		.18		1.064 (1.009-1.121)		.02	
	Mixed	0.950 (0.888-1.016)		.13		0.978 (0.914-1.045)		.51	
	Other	0.919 (0.862-0.979)		.009		0.924 (0.867-0.984)		.01	
**Grade**									
	Well differentiated	1 (reference)		N/A		1 (reference)		N/A	
	Moderately differentiated	1.594 (1.503-1.690)		<.001		1.544 (1.454-1.640)		<.001	
	Poorly differentiated or undifferentiated	2.862 (2.693-3.041)		<.001		2.272 (2.126-2.427)		<.001	
**Molecular subtype**									
	Luminal A	1 (reference)		N/A		1 (reference)		N/A	
	Luminal B	0.894 (0.849-0.941)		<.001		0.946 (0.898-0.996)		.03	
	HER2-enriched	1.164 (1.092-1.240)		<.001		1.040 (0.975-1.110)		.23	
	Triple negative	2.224 (2.139-2.313)		<.001		1.560 (1.482-1.641)		<.001	
**T (tumor size) stage**									
	T0	1 (reference)		N/A		1 (reference)		N/A	
	T1	0.735 (0.477-1.133)		.16		0.623 (0.405-0.958)		.03	
	T2	1.007 (0.655-1.548)		.98		1.136 (0.739-1.745)		.56	
	T3	1.428 (0.927-2.201)		.11		1.555 (1.011-2.393)		.045	
	T4	2.072 (1.335-3.217)		.001		2.160 (1.402-3.329)		<.001	
**N (lymph nodes) stage**									
	N0	1 (reference)		N/A		1 (reference)		N/A	
	N1	1.202 (1.132-1.276)		<.001		1.369 (1.311-1.429)		<.001	
	N2	1.618 (1.477-1.774)		<.001		1.821 (1.714-1.935)		<.001	
	N3	8.945 (8.212-9.745)		<.001		2.261 (2.093-2.442)		<.001	
**Seventh edition of the American Joint Committee on Cancer (AJCC) staging**									
	IA	1 (reference)		N/A		—^b^		N/A	
	IB	1.389 (1.196-1.612)		<.001		—		—	
	IIA	2.029 (1.882-2.188)		<.001		—		—	
	IIB	2.791 (2.493-3.124)		<.001		—		—	
	IIIA	3.399 (2.973-3.888)		<.001		—		—	
	IIIB	4.162 (3.575-4.843)		<.001		—		—	
	IIIC	8.945 (8.212-9.745)		<.001		—		—	
**Eighth edition of the AJCC pathologic prognostic staging**									
	IA	—		N/A		1 (reference)		N/A	
	IB	—		—		1.541 (1.449-1.639)		<.001	
	IIA	—		—		2.043 (1.894-2.202)		<.001	
	IIB	—		—		2.534 (2.313-2.754)		<.001	
	IIIA	—		—		2.773 (2.533-3.036)		<.001	
	IIIB	—		—		3.060 (2).735-3.425		<.001	
	IIIC	—		—		4.380 (3.872-4.955)		<.001	

^a^N/A: not applicable.

^b^Not available.

**Table 4:** Cox multivariate analysis for cancer-specific survival according to race.

Variables		Cancer-specific survival	
		Hazard ratio (95% CI)	*P* value
**White American**			
	Seventh edition of the American Joint Committee on Cancer (AJCC) anatomic staging (AS)	1.358 (1.325-1.391)	<.001
	Eighth edition of the AJCC pathological prognostic staging (PPS)	1.244 (1.223-1.265)	<.001
**Chinese**			
	Seventh edition of the AJCC AS	1.493 (1.225-1.818)	<.001
	Eighth edition of the AJCC PPS	1.242 (1.082-1.427)	.002

### Survival and Prognostic Analyses According to All Substages

**Figure 5:**
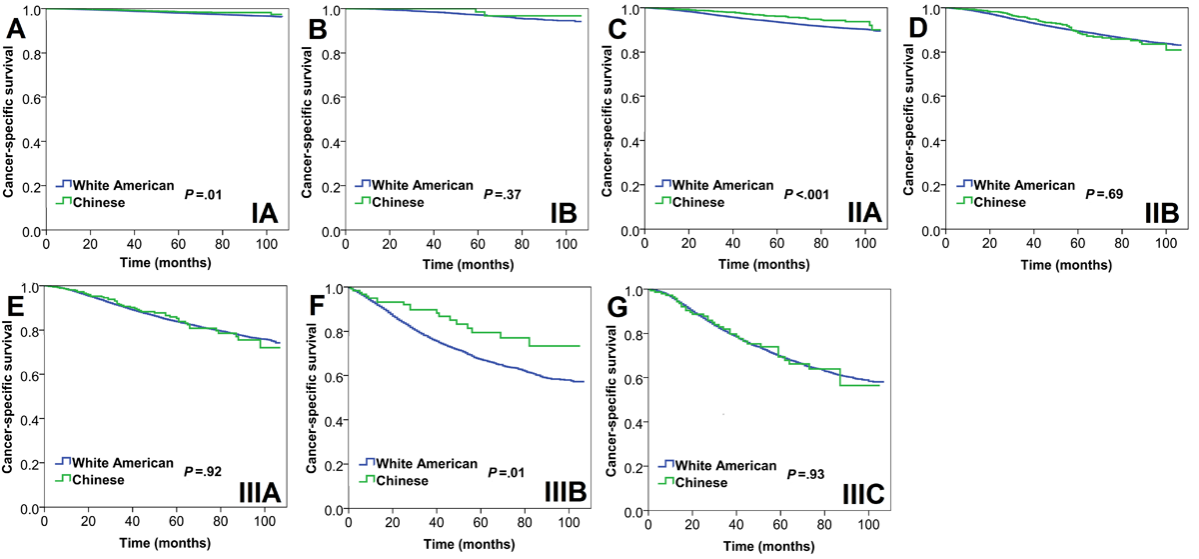
Survival curves for cancer-specific survival between White American and Chinese women with breast cancer according to the seventh edition of the American Joint Committee on Cancer substages. (A) Stage IA, (B) stage IB, (C) stage IIA, (D) stage IIB, (E) stage IIIA, (F) stage IIIB, and (G) stage IIIC.

According to the seventh AJCC AS, Chinese women had a better 5-year CSS in stage IA (98.6% vs 99.1%, *P*=.01), stage IIA (95.8% vs 97.7%, *P*<.001), and stage IIIB (78.0% vs 86.5%, *P*=.01) disease than White American women, while no significant 5-year CSS was observed in those with stage IB (98.1% vs 99.3%, *P*=.37), IIB (93.1% vs 93.8%, *P*=.69), IIIA (88.9% vs 90.7%, *P*=.92), and IIIC (78.4% vs 79.6%, *P*=.93) disease (Figure 5). Multivariate Cox proportional hazards analysis revealed that race was an independent predictor in stage IA (hazard ratio [HR] 0.679, 95% CI 0.491-0.939, *P*=.02), IIA (HR 0.683, 95% CI 0.524-0.892, *P*=.005), and IIIB (HR 0.616, 95% CI 0.392-0.969, *P*=.04) disease but not in stage IB (*P*=.56), IIB (*P*=.94), IIIA (*P*=.82), and IIIC (*P*=.70) disease (Table 5).

**Figure 6:**
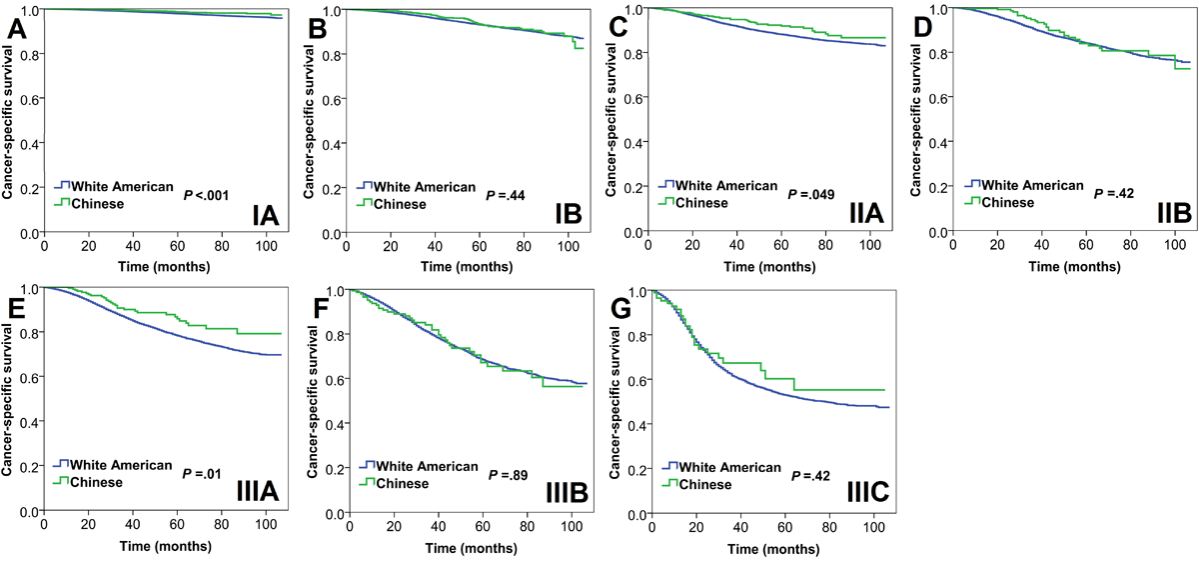
Survival curves for cancer-specific survival between White American and Chinese women with breast cancer according to the eighth edition of the American Joint Committee on Cancer substages. (A) Stage IA, (B) stage IB, (C) stage IIA, (D) stage IIB, (E) stage IIIA, (F) stage IIIB, and (G) stage IIIC.

When further stratified by the eighth AJCC PPS, Chinese women had a better 5-year CSS in stage IA (98.7% vs 99.2%, *P*<.001), stage IIA (92.0% vs 94.8%, *P*=.049), and IIIA (85.5% vs 90.8%, *P*=.02) disease than White American women, and the 5-year CSS was not significant in those with stage IB (95.6% vs 96.3%, *P*=.44), IIB (89.4% vs 91.2%, *P*=.42), IIIB (78.2% vs 78.2%, *P*=.89), and IIIC (64.5% vs 73.5%, *P*=.42) disease between White American and Chinese women (Figure 6). Cox multivariate analysis revealed that Chinese women had a better CSS in stage IA (HR 0.673, 95% CI 0.476-0.853, *P*=.002) and IIIA (HR 0.689, 95% CI 0.478-0.994, *P*=.046) disease than White American women, but race was not a prognostic factor in those with stage IB (*P*=.85), IIA (*P*=.27), IIB (*P*=.90), IIIB (*P*=.63), and IIIC (*P*=.25) disease (Table 5).

**Table 5:** Cox multivariate analysis for cancer-specific survival between White American and Chinese women according to the seventh and eighth editions of the American Joint Committee on Cancer (AJCC) substages.

Variables			Seventh edition of the AJCC staging				Eighth edition of the AJCC staging		
			Hazard ratio (95% CI)		*P* value		Hazard ratio (95% CI)		*P* value
**Stage IA**									
	White American	1 (reference)		N/A^a^		1 (reference)		N/A	
	Chinese	0.679 (0.491-0.939)		.02		0.637 (0.476-0.853)		.002	
**Stage IB**									
	White American	1 (reference)		N/A		1 (reference)		N/A	
	Chinese	0.659 (0.163-2.663)		.56		0.975 (0.750-1.268)		.85	
**Stage IIA**									
	White American	1 (reference)		N/A		1 (reference)		N/A	
	Chinese	0.683 (0.524-0.892)		.005		0.872 (0.620-1.142)		.27	
**Stage IIB**									
	White American	1 (reference)		N/A		1 (reference)		N/A	
	Chinese	1.010 (0.790-1.292)		.94		0.976 (0.675-1.410)		.90	
**Stage IIIA**									
	White American	1 (reference)		N/A		1 (reference)		N/A	
	Chinese	1.034 (0.780-1.372)		.82		0.689 (0.478-0.994)		.046	
**Stage IIIB**									
	White American	1 (reference)		N/A		1 (reference)		N/A	
	Chinese	0.616 (0.392-0.969)		.04		1.083 (0.781-1.501)		.63	
**Stage IIIC**									
	White American	1 (reference)		N/A		1 (reference)		N/A	
	Chinese	1.066 (0.769-1.477)		.70		0.790 (0.528-1.183)		.25	

^a^N/A: not applicable.

## Discussion

### Principal Findings

This study aimed to evaluate stage-specific survival in BC between Chinese and White American women in accordance with the eighth AJCC PPS. Our results show that the eighth AJCC PPS had a similar discriminating ability between White American and Chinese participants with BC compared with the seventh AJCC AS. Our study provides additional data on the use of new PPS in different races based on current real-world practices.

Advances in molecular biomarkers (ER, PR, and HER2) and their close relationship with treatment responses and prognosis rendered the traditional AS unable to meet the trend of individualized treatment [6,7,17]. The eighth AJCC PPS, which integrates the aforementioned biomarkers and grade, facilitates more precise prognosis prediction than the seventh AJCC AS [8-10]. However, the small sample size and treatment heterogeneity in their studies limited the application of the eighth AJCC PPS in BC. In our real-world study with a large sample size (n=376,818), the eighth AJCC PPS revealed better prognostic accuracy than the seventh AJCC AS (*P*<.001) and performed well with discriminating ability consistent with disease stages. Therefore, the new AJCC staging system could better predict prognosis and guide the treatment of BC.

Our study shows that 149,452 (39.7%) individuals with BC migrated from the AJCC AS to AJCC PPS, which was similar to the rates observed in previous studies (20.7%-52.8%) [10,18-22]. The downstaging rate was significantly higher than upstaging rate (33.7% vs 6.0%) in this study, and the results are consistent with those of previous studies (downstaging: 15.2%-42.1%; upstaging: 5.5%-41.0%) [10,18-22]. Change in stage leads to diverse therapeutic decisions. The new AJCC staging enabled 126,936 (33.7%) participants to be downstaged, and these patients might be exempt from the therapies, such as chemotherapy and radiotherapy, which could ensure efficacy and reduce the treatment burden of patients [23]. In our previous studies, we found that the new AJCC staging can accurately guide individualized treatment of patients with BC in clinical decision-making. Patients who were downstaged from the eighth AJCC PPS can safely avoid adjuvant chemotherapy or radiotherapy [24-26]. Therefore, the 8th AJCC staging better reflects the trend of personalized treatment. In addition, among the patients with stage changes in this study, White American participants had a higher upstaging rate than Chinese participants (6.0% vs 5.3%), while the latter had a higher downstaging rate than the former (35.2% vs 33.6%). The Will Rogers phenomenon might explain the differences in “stage migration” in individuals with cancer who are of different races. Differences in culture, education, and diet lead to differences in migration rates between different ethnic groups [27-29]. In addition, socioeconomic status might be another critical factor affecting stage distribution and survival outcomes in different races. A study by Kantor et al [30] included 259,852 individuals with BC who are of different races and reported that non-Hispanic Black individuals and those of lower socioeconomic status had a lower disease-specific survival, even in all substages of the PPS [30].

The initial model for establishing the new AJCC PPS in BC was based on 305,519 patients from National Cancer Database between 2010 and 2012 [11]. However, the majority of the participants were White American and Hispanic American [11]. Therefore, the applicability of the eighth edition of the AJCC staging in Asian individuals, especially in Chinese individuals, remains unclear. Several retrospective studies explored the value of new AJCC staging in Chinese individuals with BC. However, only partial subgroups of BC, such as T1-2N1 and TNBC were included [13-15,31]. The cohort study conducted by He et al [15] recruited patients with TNBC from Sun Yat-sen University Cancer Center (n=611) and the SEER database (n=31,941) to examine the prognostic value of the eighth AJCC PPS in comparison with the seventh AJCC AS. However, no significant discriminatory ability was observed between the 2 staging systems in Chinese individuals with BC in this study and patients from the SEER database [15]. The opposite result was obtained in another study conducted by Yang et al [31], which included 1556 Chinese individuals with BC and compared the prognostic value of the 2 staging systems. They found that the new AJCC PPS had better accuracy of prognosis prediction than AS in Chinese individuals with BC [28]. However, their sample size was relatively small, especially in the eighth AJCC PPS of stage IIB (n=83) and IIIC (n=22). Therefore, their result might not accurately reflect the value of new staging [31]. In addition, most of the studies assessing the effect of the eighth AJCC PPS in the Chinese population lacked a comparison with the standard population, and their applicability may not be adequate [13-15,31]. In our study, we used a much larger sample to evaluate the new AJCC staging, and we observed a better discriminating value than that of the AJCC AS regardless of race. Therefore, our study better verified the applicability of the new staging in Chinese individuals with BC.

In a previous SEER study, Lim et al [4] reported that Chinese women with BC in the United States have better CSS than White American women, and the largest survival differences between Chinese and White American women were observed for stage I and node-negative cancers [4]. In this study, using the new AJCC staging, we found that Chinese women had superior CSS among those with stage IA and stage IIIA disease compared to White American women. The main reasons for this difference are not clear. The differences in treatment compliance and inherent genetic predisposition may lead to differences in survival between the 2 ethnic groups [32-35].

### Limitations

There are several limitations to be acknowledged in this study. First, we extracted the patient data from the SEER database, and selection biases inherently existing in retrospective studies should not be disregarded. Second, although the sample size of the group of Chinese individuals was much larger than that in previous studies, the number of individuals in some substages, such as stage IIIC (n=87), was still small. Therefore, the value of new AJCC staging in Chinese individuals with BC should be further explored. Third, details of treatment were not collected in the SEER database, including radiotherapy (technique, target volume, and radiation dose), chemotherapy regimens, endocrine therapy (regimen and duration), and targeted therapy, which may potentially affect the final analysis. Even with these limitations, our study reflects real-world practices and extends the applicability of the new staging.

### Conclusions

In conclusion, our study suggests that the eighth AJCC PPS has a similar discriminating ability in White American and Chinese individuals with BC than the AJCC AS. Therefore, the new staging is also applicable to Chinese individuals with BC. Further studies are needed to explore the value of the PPS in Chinese individuals with BC.

## Abbreviations

AJCC: American Joint Committee on Cancer
AS: anatomic staging
AUC: area under the curve
BC: breast cancer
CSS: cancer-specific survival
ER: estrogen receptor
HR: hazard ratio
M: distant metastasis
N: lymph nodes
PPS: pathological prognostic staging
PR: progesterone receptor
SEER: Surveillance, Epidemiology, and End Results
T: tumor size
TNBC: triple-negative breast cancer
